# Fear and stock price bubbles

**DOI:** 10.1371/journal.pone.0233024

**Published:** 2020-05-12

**Authors:** Thorsten Lehnert

**Affiliations:** Department of Finance, University of Luxembourg, Luxembourg, Luxembourg; Institute for Economic Forecasting, Romanian Academy, ROMANIA

## Abstract

I evaluate Alan Greenspan’s claim that stock price bubbles build up in periods of euphoria and tend to burst due to increasing fear. Indeed, there is evidence that e.g. during a crisis, triggered by increasing fear, both qualitative and quantitative measures of risk aversion increase substantially. It is argued that fear is a potential mechanism underlying financial decisions and drives the countercyclical risk aversion. Inspired by this evidence, I construct an euphoria/fear index, which is based on an economic model of time varying risk aversion. Based on US industry returns 1959–2014, my findings suggest that (1) Greenspan is correct in that the price run-up initially occurs in periods of euphoria followed by a crash due to increasing fear; (2) on average already roughly a year before an industry is crashing, euphoria is turning into fear, while the market is still bullish; (3) there is no particular euphoria-fear-pattern for price-runs in industries that do not subsequently crash. I interpret the evidence in favor of Greenspan, who was labeled “Mr. Bubble” by the New York Times, and who was accused to be a serial bubble blower.

“*Fear and euphoria are dominant forces*, *and fear is many multiples the size of euphoria*. *Bubbles go up very slowly as euphoria builds*. *Then fear hits*, *and it comes down very sharply*. *When I started to look at that*, *I was sort of intellectually shocked*. *Contagion is the critical phenomenon which causes the thing to fall apart*.*”*

Alan Greenspan

## Introduction

The distinguished economist and former chairman of the Federal Reserve Alan Greenspan does believe that security prices exhibit price bubbles. A price bubble can be defined as an irrational strong price increase, followed by a remarkable crash in the price of the security. Greenspan argues that the price run-up is fueled by euphoria in the market. When euphoria turns into fear, security prices are going to crash. Importantly, such a bubble in stock prices would not be predictable ex ante but observable ex post. However, price bubbles could exhibit the particular euphoria-fear pattern that Greenspan describes if bubbles build up endogenously when the market economy evolves. But is there evidence for that?

In light of the recent price bubble analysis of Greenwood et al. [1], I seek to address this question in this paper. I propose a method that relies on an economic model of time varying risk aversion to distinguish periods of euphoria and fear. There is evidence that during a crisis both qualitative and quantitative measures of risk aversion increase substantially. Researchers argue that fear is as a potential mechanism underlying financial decisions and drives the countercyclical risk aversion (see Guiso et al. [2] and Cohn et al. [3]). Inspired by this evidence, I construct an euphoria index, where periods of euphoria (fear) are associated with low (high) levels of relative risk aversion. I make use of NBER recession periods and historic S&P 500 bear markets in order to evaluate the relevance of this index. The Greenwood et al. [1] methodology is used to identify periods of price run-ups and subsequent crashes for US industries. In line with Greenspan’s arguments, I hypothesize that periods of price-run-ups can be associated with periods of euphoria and periods of crashes can be associated with periods of fear.

The rest of the paper is structured as follows: In section 2, I review the literature. I discuss the data and present the methodology in section 3. In section 4, we present the empirical results of the study and section 5 concludes.

### Fear and risk aversion

The view that risk aversion is time-varying is well accepted in the literature. Campbell and Cochrane [4] formulate a consumption-based asset pricing model in which news about consumption growth (as in a habit formation model) leads to time variation in investors risk aversion. Brandt and Wang [5] extend the model and additionally allow risk aversion to change over time in response to news about inflation, which leads to a different interpretation. From a rational perspective, their model can be interpreted as a reduced form of an economy with time-varying risk aversion. Inflation is used as a proxy for the unobserved effects of a neglected friction. Moreover, from a behavioral standpoint, the representative agents in the Brandt and Wang model exhibits an irrational fear for an unexpected inflation shock.

During his 1933 first inaugural address, in the depth of the Great Depression, Franklin Roosevelt famously said, “The only thing we have to fear is fear itself”. Hence, emotional reactions to risky situations often diverge from cognitive assessments of those risks. When faced with a negative shock, individuals are affected by an emotion (fear) that alters their willingness to take risk in both financial and non-financial domains (Loewenstein [6]). Lerner and Keltner [7–8] propose an appraisal-tendency framework that links emotion-specific appraisal processes to a broad array of judgment and choice outcomes. They find that, in particular, fear is strongly related to risk perception. In their experimental set-up, fearful people express pessimistic risk estimates and make more risk-averse choices. In their “Depression Babies” paper, Malmendier and Nagel [9] ask whether people who live through different macroeconomic histories make different risky choices. They present supporting evidence that households’ risk-taking decisions are strongly related to the lifetime average return and inflation. Hence, their findings suggest that the causality seems to go from personal experiences of macroeconomic shocks to investors’ willingness to bear financial risk. Cohn et al. [3] experimentally test if investors exhibit countercyclical risk aversion. They prime financial professionals with either a boom or a bust scenario and subsequently measure their risk aversion. Subjects who were primed with a financial bust were substantially more risk averse (more fearful) than those who were primed with a boom. Furthermore, they find that fear is negatively related to investments in the risky asset, suggesting that fear drives the countercyclical risk aversion. The findings of Guiso et al. [2] suggest that, during the crisis, both qualitative and quantitative measures of risk aversion increase substantially and that affec individuals divest more from stock. They identify fear as a potential mechanism underlying financial decisions. Fear leads to an increase of the curvature of the utility function or the salience of negative outcomes. Their evidence suggests that fear relates to periods of high risk aversion.

### Bubbles

There is a lot of research in finance on rational and irrational bubbles (see Kindleberger [10]; Garber [11–12]; Pastor and Veronesi [13–14]; Greenwood and Nagel [15] and the theoretical papers of De Long et al. [16], Abreu and Brunnermeier [17], Scheinkman and Xiong [18], and Barberis, Greenwood, Jin, and Shleifer [19]). The rational expectations school understands bubbles as the intelligent work of the invisible hand (Blanchard and Watson [20]; Tirole [21] and Diba and Grossman [22]). Rational asset pricing helps to understand high prices during bubble incidents due to forecasts in cash flows (Garber [11]), changes in discount rates (Pastor and Stambaugh [23–24]) and a mixture of risk premia and learning (Pastor and Veronesi [13–14]). Pastor and Veronesi [13] propose a general equilibrium model in which stock prices of innovative firms exhibit bubbles during technological revolutions. They show that the nature of the risk associated with new technologies changes over time. Due to the small scale of production and a low probability of a large-scale adoption, the risk is initially mostly idiosyncratic. It remains idiosyncratic for those technologies that are never adopted on a large scale. Once a technology is ultimately adopted, the risk must gradually change from idiosyncratic to systematic. As the probability of adoption increases, the new technology becomes more likely to affect the old economy and with it the representative agent’s wealth. The resulting increase in systematic risk depresses stock prices in both the new and old economies, because it pushes up the discount rates. Obviously, because the new economy discount rates rise higher due to an increase in the new economy’s market beta, their stock prices fall deeper. Importantly, such a rational bubble in stock prices would be observable ex post but unpredictable ex ante. In the spirit of Alan Greenspan, one could hypothesize that with the increasing probability of adaption of the new technology, ‘a bubble goes up slowly as euphoria builds’. When systematic risk increases, ‘fear hits and it comes down very sharply’.

In contrast to the rational expectations school, Galbraith sees bubbles as a recurring loop of delusion built-up by the market mechanism, but as fundamentally irrational and due to mass euphoria, herd behavior and greed (Galbraith [25]). Along the same lines, Perez [26] argues that the two recent boom and busts—the internet mania and crash around the year 2000 and the easy liquidity boom and bust around the Lehman default in 2008—are two distinct components of a single structural phenomenon; the first was based on technological innovation, the second on financial innovation. She claims that those ‘episodes are endogenous to the way in which the market economy evolves and assimilates successive technological revolutions’. Bubbles start building up when investors switch from seeking dividends to pursuing capital gains and the decoupling from the real economy is taking place. The subsequent collapse leads to a reconnection with the real economy and the start of a period when control passes over from financial capital to production capital.

The technological revolution/financial innovation story is somewhat in line with Alan Greenspan’s claim about euphoria and fear, but the causal relationship between euphoria and bubbles and its link with fundamentals is not obvious. A well-known characterization of euphoria and its relation with bubbles is the one of Minsky, who distinguishes between several phases (see e.g. Kindleberger [10] or Brunnermeier and Oehmke [27]). The first phase is an early period, the initial displacement. A new technology or financial innovation leads to expectations of increased profits and economic growth. The resulting boom phase is usually characterized by credit expansion, low volatility, and more investments. The boom period is characterized by rising asset prices with growing momentum. Prices start exceeding the actual fundamental improvements from the innovation and, fueled by euphoria, investors start trading the overvalued asset in a frenzy. Concerned about increased price volatility (and increasing fear), institutional investors start taking their profits, but there is still enough demand from less sophisticated investors such that prices are still rising. Finally, a trigger event leads to the bursting of the bubble, typically called the “Minsky moment”, which has built up in the background. Prices are collapsing, because investors dump the asset, which leads to a panic with spill-over effects and severe overshooting in the downturn.

Several theoretical models explain how volatility can increase as a bubble builds. For example, Scheinkman and Xiong [18] propose an equilibrium model where overconfidence generates disagreements among agents regarding asset fundamentals. These disagreements, combined with short-sale constraints, can push up both the asset price and its volatility. In a series of papers, Bekaert et al. [28–31] aim to disentangle risk aversion (or its inverse, which they call “risk appetite”) and the uncertainty about fundamentals. They identify the relative importance of changes in the conditional variance of fundamentals and changes in risk aversion in the determination of the risk premiums. Their findings suggest that variation in the equity premium is driven by both risk and uncertainty with risk aversion dominating. However, variation in asset prices is primarily due to changes in risk. Branch and Evans [32] propose an asset-pricing model that generates bubbles and crashes. Agents use learning to forecast expected returns and the conditional variance of stock returns. Risk aversion and uncertainty regarding the riskiness of the asset generate changes in beliefs as a result of fundamental shocks, which leads to price run-ups. Agents’ risk aversion increases over time and leads to a bursting of the bubble. Hence, aversion to risk and adaptive learning can generate bubbles, run-up periods with subsequent crashes. They show that an aversion to risk is critical for the build-up, as well as the bursting of a bubble.

During bubbles, the real economy and the financial economy are obviously decoupled. A bubble takes root in a particular segment, e.g. new technology stocks, but then disregards the fundamental and uses these stocks as mere objects of price-change. There are similar examples for tulips, gold or housing markets. For example, in the 1990s bubble, the price of the whole stock market exceeds forty times the previous decade’s average earnings, with dividends being smaller or even non-existent. This can be seen as an extraordinarily high estimate of future earnings, which cannot be true for many stocks, or the expectation of even higher prices bringing quick capital gains. Essential parts of feedback processes that inflate the bubble are both channels (Perez [26]). The run-up and crash periods cannot be seen in isolation, they are two sides of the same coin. In my paper, I aim to find a pattern in fear/euphoria that helps to identify at an early stage the existence of a bubble (a run-up period with a subsequent crash) vis à vis a ‘normal’ run-up period (without a subsequent crash.) My proxy for euphoria is based on an economically sound measure that serves as a proxy for the irrational fear of a representative agent to experience an unexpected inflation shock. In the US, high expected inflation typically coincides with periods of heightened uncertainty about real economic growth and unusually high risk aversion, both of which raise equity yields. I hypothesize that, after an initial phase of euphoria, this increasing fear is critical for building, as well as popping of a bubble. Hence, I assume that the euphoria/fear pattern relates to actual bubbles, not to simple run-up episodes.

More recently, Greenwood et al. [1] evaluate Eugene Fama’s claim that stock prices do not exhibit price bubbles. Their findings suggest that at the industry level, on average, substantial price increases cannot be predicted to be succeeded by unusually low returns. Nevertheless, observing those boom episodes of industry portfolios significantly increase the probability of a subsequent crash. Investors can time the bubble by relying on some of the characteristics of the price run-up. In the empirical part of the paper, I make use of their methodology to identify industry price run-ups and crashes.

## Materials and methods

Cochrane and Campbell [4] propose a consumption-based asset pricing model in which time variation in investors risk aversion is imposed by news about consumption growth (as in a habit formation model). Brandt and Wang [5] extend the model by additionally allowing risk aversion to change over time in response to news about inflation. Relying on an endowment economy, they formulate a model, where an infinitely lived representative agent maximizes the conditional expectation of lifetime utility of consumption

$$u(C_{0},C_{1},C_{2},\ldots ,C_{\infty })=\sum_{t=0}^{\infty }\delta^{t}u(C_{t}-X_{t})$$ (1)

where

$$u(C_{t}-X_{t})=[\begin{array}{cc} ln(C_{t}-X_{t}) & if\;\alpha =1\\ \frac{{\left(C_{t}-X_{t}\right)}^{1-\alpha }-1}{1-\alpha } & if\;\alpha >0\;and\;\alpha \neq 1 \end{array}$$ (2)

Here *δ* is a subjective discount factor, and *α* measures the curvature of the utility function. Furthermore, *C*_*t*_ is the level of consumption and *X*_*t*_ is the subjective reference level, a habit, at time *t*.

In this setup a few points are important. Firstly, the consumption is always higher than the habit level, so that *C*_*t*_−*X*_*t*_>0, and the utility function is measurable. Secondly, the habit is in the steady state not affected by consumption $\left(\frac{dX_{t}}{dC_{t}}=0\right)$. And finally, with any increase in the consumption the habit should increase $\left(\frac{dX_{t}}{dC_{t}}>0\right)$, except in the steady state. In this case, the Relative Risk Aversion at time *t* (*RRA*_*t*_) will be

$${RRA}_{t}=-\frac{C_{t}u^{\prime \prime }(C_{t}-X_{t})}{u^{\prime }(C_{t}-X_{t})}=\alpha \frac{C_{t}}{C_{t}-X_{t}}$$ (3)

Hence following Brandt and Wang (2003), I define the dynamic of log relative risk aversion *γ*_*t*_ = ln(*RRA*_*t*_) as

$$\gamma_{t+1}=\bar{\gamma }+\emptyset (\gamma_{t}-\bar{\gamma })-e_{t+1}$$ (4)

where

$$e_{t+1}=\lambda (\gamma_{t})(g_{t+1}-E_{t}[g_{t+1}])-\theta (\gamma_{t})(\pi_{t+1}-E_{t}[\pi_{t+1}])$$ (5)

The investor risk aversion is mean-reverting if ∅<1. In (Eq 5), *g*_*t*+1_ = *ln*(*C*_*t*+1_)−*ln*(*C*_*t*_) is the log consumption growth rate and *π*_*t*+1_ = *ln*(*CPI*_*t*+1_)−*ln*(*CPI*_*t*_) denotes the log inflation. Furthermore, *λ*(*γ*_*t*_) reveals the sensitivity of relative risk aversion to news about consumption growth and *θ*(*γ*_*t*_) reveals the sensitivity of relative risk aversion to news about inflation. Hence, unexpected consumption growth and disinflation represent good news when sensitivities are positive, which leads temporarily to lower aggregate risk aversion. Following Brandt and Wang [5], I set the sensitivities as

$$\lambda (\gamma_{t})=\frac{1}{\alpha }\exp\left(\gamma_{t}\right)-1\;\mathrm{and}\;\theta (\gamma_{t})=\theta \frac{1}{\alpha }\exp\left(\gamma_{t}\right)-1$$ (6)

(Eq 6) suggests that an increase in risk aversion increases the responsiveness of an investor to news in consumption growth. *g*_*t*_ and *π*_*t*_ are assumed to follow a VAR process and the volatility of consumption growth is assumed to be constant over time, while the conditional volatility of inflation is assumed to follow an asymmetric GARCH model (see Brandt and Wang [5] for details). There is limited evidence that the volatility of consumption growth is varying over time. However, when we allow the volatility of consumption growth to be time-varying, results qualitatively don’t change.

Hence, in this model, the price $P_{t}^{n}$ of an n-period default-free nominal discount bond is $P_{t}^{n}=E_{t}[M_{i,t+n}]$, where *M*_*i*,*t*+*n*_ is the nominal pricing kernel:

$$M_{t,t+n}=\delta^{n}exp\left(\alpha (\gamma_{t+n}-\gamma_{t}-(g_{t+1}+\cdots +g_{t+n}))-(\pi_{t+1}+\cdots +\pi_{t+n})\right)$$ (7)

Through recursive expressions, one can obtain the prices of equity claims:

$$S_{t}=E_{t}[M_{t,t+n}(S_{t+n}+D_{i,t+n})]$$ (8)

where *S*_*t*_ is the nominal stock price and *D*_*i*,*t*+*n*_ denotes the sum of nominal dividends payed on the stock between dates t and t+n. The nominal stock return *R*_*t*,*t*+*n*_ = (*S*_*t*+*n*_+*D*_*t*,*t*+*n*_)/*S*_*t*_ has to satisfy 1 = *E*_*t*_[*M*_*t*,*t*+*n*_ *R*_*t*,*t*+*n*_].

In a first step, the structural parameters of the VAR process are estimated through maximum likelihood. In the second step, I estimate the preference parameters $\alpha ,\bar{\gamma },\emptyset ,\theta ,\delta$ through the Generalized Method of Moments (GMM) with stock returns. One major building block of the model is the dynamics of the two exogenous state variables. Previous research suggest that the co-dependents of consumption and inflation is important. Following Boudoukh [33], among others, I assume that consumption growth and inflation follow a VAR-SV type process. The author shows that the mean, variance, and autocorrelation of yields is captured relatively well by such a model, calibrated with inflation and consumption data. The co-dependents of consumption and inflation are found to be important determinants for both real and nominal rates. The residuals from the first-step regression (the estimation of the structural parameters) are then used in the second-stage estimation of the preference parameters. Obviously, the two-step procedure has computational advantages and avoids the problem of overfitting asset prices and returns, because of unreasonable dynamics of the exogenous state variables.

I expand the set of nominal moments with a vector of conditioning instruments *Z*_*t*_.

$$1{⊗Z}_{t}=E_{t}[M_{t,t+n}(\Theta )R_{t,t+n}{⊗Z}_{t}],$$ (9)

where ⊗ denotes the Kronecker product. By applying the law of iterated expectations to obtain a set of unconditional moments *E*[*h*_*t*+1_(Θ)] = *E*[(*M*_*t*,*t*+1_(Θ)*R*_*t*,*t*+1_−1)⊗*Z*_*t*_] = 0, I construct a GMM estimator and find Θ that minimize the norm of *h*_*t*+1_(Θ)

$${\left[\frac{1}{T}\sum_{t=1}^{T}h_{t+1}(\Theta )\right]}^{\prime }W_{T}\left[\frac{1}{T}\sum_{t=1}^{T}h_{t+1}(\Theta )\right]$$ (10)

*W*_*T*_ is the optimal weight matrix that I update in each iteration of the optimization process (Hansen [34]).

Therefore, in the model aggregate risk aversion is time-varying in response to both news about consumption growth (as in a habit formation model) and news about inflation, which leads to a rather general framework for modeling time-varying risk aversion. From a rational perspective, the model can be interpreted as a reduced form of an economy with time-varying risk aversion. Inflation is used as a proxy for the unobserved effects of a neglected friction. Moreover, from a behavioral standpoint, the representative agents in the Brandt and Wang model exhibits an irrational fear for an unexpected inflation shock. In a related study, Malmendier and Nagel [9] argue that households’ risk-taking decisions are strongly related to experiences of macro-economic outcomes, the lifetime average return and inflation. Hence, their findings suggest that the causality seems to go from personal experiences of macroeconomic shocks to investors’ willingness to bear financial risk. Subsequently, I make use of this interpretation and use the time-varying risk aversion as a proxy for aggregate fear among agents in the economy and construct a market-wide index of fear and euphoria.

## Results and discussion

### Time-varying risk aversion

I select the dividend yield, default premium, term premium and 1-month US Treasury yield as the conditioning variables, and fit the model to the monthly time series of 25 Fama and French size and book-to-market sorted stock portfolios. Brandt and Wang [5] also estimate the preference parameters with bonds, as well as with bonds and stocks. They find that that the resulting time series of risk aversion is counter-cyclical and more volatile when the model is only fitted to stock data. I calculate the investor risk aversion for a period from 1959 to 2014.

The remarkable cross-sectional dispersion in expected returns between the 25 Fama and French stock portfolios and our long study period, with several economic expansions and recessions, enables us to compute the preference parameters and, hence, the relative risk aversion time series accurately. Estimated values are significant at the 5% level and economically plausible, where standard errors are autocorrelation and heteroscedasticity adjusted. The estimate of θ, the parameter that measures the sensitivity of log relative risk aversion to news about inflation, is 24.32, which implies that, given our specification of the sensitivity function, the steady-state effect of an inflation shock is substantially larger than a comparable consumption growth shock. The estimates of *α* and $\bar{\gamma }$ are 1.65 and 1.14, respectively, and are both somewhat larger compared to the Brandt and Wang (2003) results, which typically translates into a higher average level of risk aversion. The other estimates (*δ* = 0.994 and ∅ = 0.95) are considered reasonable in the literature and suggests that risk aversion varies only gradually through time. The model generates plausible time variation in aggregate risk aversion. Risk aversion ranges from 1.86 to 5.46, with an average level of 3.07. This is reasonable interval based on the past literature. As a comparison, Brandt and Wang (2003) find that when fitted to stock data, relative risk aversion ranges from 2.10 to 6.93 in their sample. Risk aversion is highly persistent, with an autocorrelation of 0.94. Aggregate risk aversion is known to be counter-cyclical, therefore, fluctuating over the business cycle, high and rising in recessions and low and falling in expansions (Fama [35]). Given that inflation and real returns are countercyclical indicators of economic conditions, I expect to find a positive correlation. I find the correlation with inflation to be 0.47, suggesting that high inflation can be associated with high aggregate risk aversion (see Shiller [36]). I also find a positive correlation with the real returns on a 1-month Treasury bill (0.22), implying that in times of high risk aversion, the real return is also high, driving down the future level of risk aversion.

### Euphoria index

**Fig 1:**
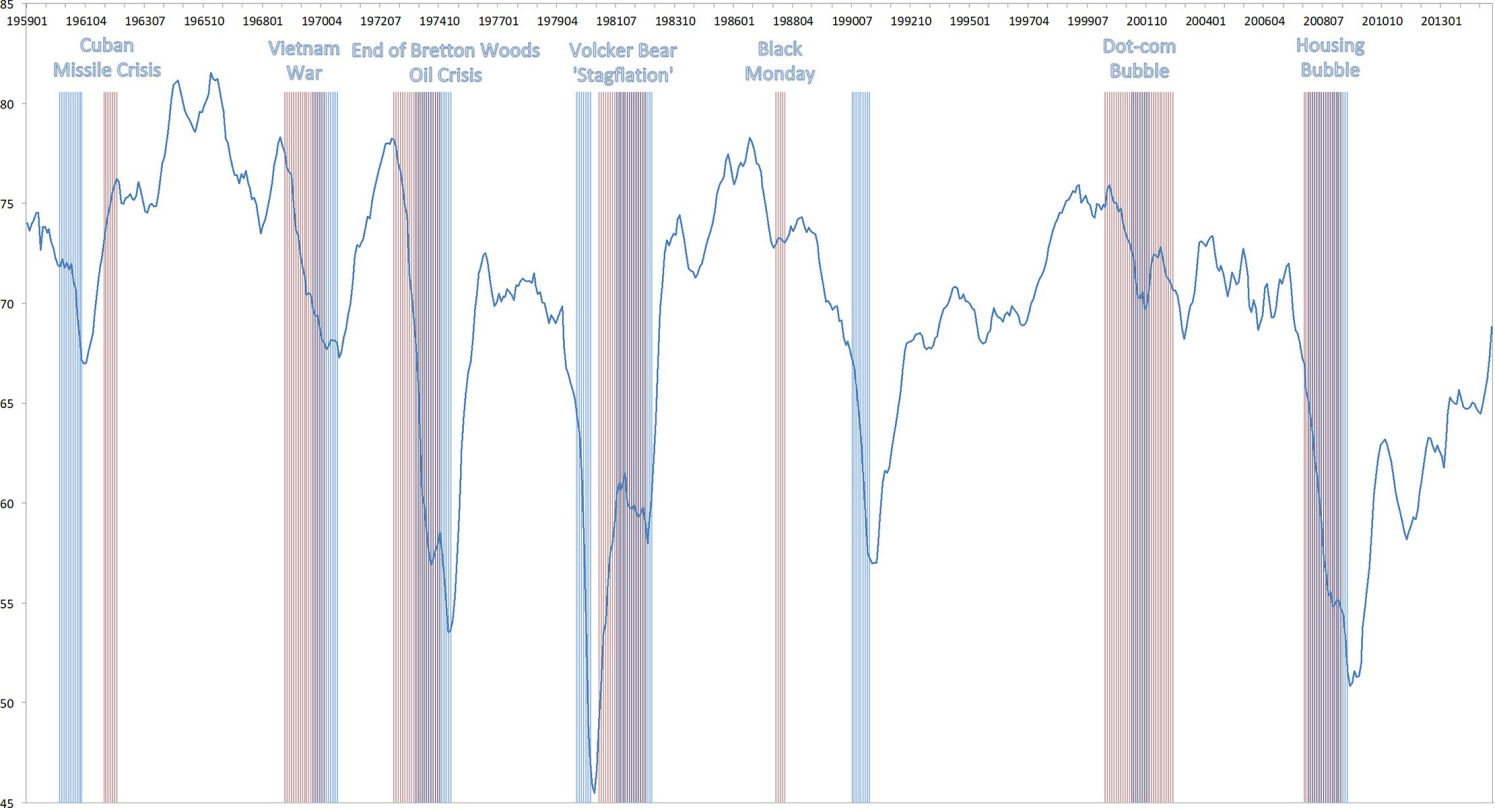
Euphoria Index, S&P 500 bear markets and NBER recession periods. The Figure depicts the level of the Euphoria Index for a time period 1959 until 2014. The highlighted areas present the NBER recession periods (in blue) and the S&P 500 bear markets (in red). For each of the historic bear markets, I indicate the source of the overall pessimism on Wall Street in that period.

Inspired by previous evidence, I construct an euphoria index (EIx), where periods of euphoria (fear) are associated with low (high) levels of relative risk aversion. The derivation is similar to the construction of the CBOE SKEW index. The CBOE SKEW index is derived from the price of S&P 500 index option-implied skewness, denoted by S. S is calculated from a portfolio of S&P 500 options that mimics an exposure to a skewness payoff. Since S tends to be negative and to vary within a narrow range (e.g. -4.69 to—0.10 between 1990 and 2010), it is inconvenient to use it as an index. S is therefore transformed to SKEW by the following linear function: SKEW = 100–10 * S. With this definition, SKEW increases as S becomes more negative and tail risk increases. Hence, the relative risk aversion is transformed to an index EIx by the following linear function: EIx = 100–10 * RRA. With this definition, EIx decreases as the relative risk aversion in the market increases. The index is able to differentiate between periods of fear and euphoria. There is evidence that broad stock market price indices tend to fall steeply before and during recessions. Investors are more risk-averse and thus demand a higher return for holding stocks. Campbell and Cochrane’s [34] habit formation model suggest that when the economy is about to go into recession, investors have fewer resources to maintain their living standards. Hence, they are less willing to bear financial risk. The expected equity premium needs to go up in order for them to still hold stocks. Hence, since dividends are discounted by a higher rate, stock prices fall during recessions. Lettau and Ludvigson [37] provide some empirical evidence for this explanation. In the following, I compare the EIx with notable recession periods. NBER recession periods are: December 1969 –November 1970, December 1969—November 1970, November 1973—March 1975, January 1980—July 1980, July 1981—November 1982, July 1990—March 1991, March 2001—November 2001 and December 2007—June 2009. Furthermore, the crashes that I identify can be industry-specific, therefore, they do not necessarily have a relationship with a broad, aggregate euphoria/fear index. Hence, in Fig 1, the euphoria-fear-index is plotted together with the NBER recession periods (in blue) and historic bear markets (in red). By one common definition, a bear market occurs when stock prices (the S&P500 market index) fall for a sustained period, dropping at least 20 percent from their peak (see ‘Global Financial Data’, msnbc.com research, www.nbcnews.com or "History of bear markets since 1929" by Jade Scipioni, published December 26, 2018, www.foxbusiness.com). Historic bear markets based on the S&P 500 are found to relate to the following time periods: December 1961 to June 1962 (Cuban Missile Crisis, S&P 500 loss: 28 percent), November 1968 to May 1970 (Vietnam War, S&P 500 loss: 36 percent), January 1973 to October 1974 (End of Bretton Woods–Oil Crisis, S&P loss: 48 percent), November 1980 to August 1982 (Volcker Bear–Stagflation, S&P 500 loss: 27 percent), August 1987 to December 1987 (Black Monday, S&P 500 loss: 34 percent), March 2000 to October 2002 (Dot-com Bubble, S&P 500 loss: 49 percent), October 2007 to March 2009 (Housing Bubble, S&P 500 loss: 57 percent). For each of the historic bear markets, I indicate the source of the overall pessimism on Wall Street in that period.

In line with the theoretical reasoning, results suggest that NBER recession periods are preceded by periods of increasing fear. Furthermore, fear further increases during recessions. In the subsequent stable periods or periods of expansion, euphoria increases substantially. Evidently, bear markets are strongly related to and oftentimes preceded by periods of increasing fear. Except for the bear market in the early 60’s around the Cuban Missile Crisis, the level of the EIx is typically low or decreasing, when the stock market is doing poorly. Not surprisingly, bear markets are also overlapping with periods of recessions. Hence, the findings suggest that the EIx is a reasonable proxy to evaluate Greenspan’s claim.

### Crashes after price run-ups

Industry run-ups and crashes are identified by replicating the analysis of Greenwood et al. [1]. Based on US industry data, I isolate all episodes in which an industry experiences value-weighted raw returns and a net-of-market return of more than 100% in the past two years, as well as more than 50% raw return over the past five years. As the authors correctly point out, the 2- and 5-year horizon is important, because one wants to avoid picking up recoveries from periods of poor performance. In line with Greenwood et al. [1], I replicate their analysis using stock data and including recently listed firms. Using the most recent SIC code on Compustat, or CRSP, stocks are matched to industries each month and returns are value-weighted. Industry portfolios are ideal for this type of analysis. In terms of statistical power of the analysis, analyzing industries is preferable over analyzing the entire stock market. Furthermore, historically, booms and busts have a strong industry component, e.g. the stock market boom of the late 1990s was concentrated in internet stocks (Ofek and Richardson [38]). In order to make use of the EIx in this exercise, my sample includes returns from January 1959 to December 2014, which allows us to identify 2-year run-up periods and a subsequent 2-year evaluation period over a 55 years period. A two-year price path afterwards is required to classify industries that crash and that experience a further run-up. In line with Greenwood et al. [1], a crash is defined as a 40 percent or more drawdown in absolute terms beginning at any point within the 2-year evaluation period after the price increase was identified for the first time. I also use net-of-market returns for the run-up period in order “to avoid classifying as a potential bubble an industry with modest or flat performance during a time when the market performed poorly”. During my sample period, in total 30 run-up episodes can be classified into 15 that crash in the subsequent two years and 15 that do not crash. Using those criteria, I am able to perfectly replicate the results of Greenwood et al. [1]. Industry crashes and non-crashes are presented in Tables 1 and 2.

**Table 1:** Industry price run-ups–crashes.

	Run-up period			Subsequent 2-years		
*S&P 500 Bear Market* Industry Name	Run-up Start	Run-up End	Number of Firms	Price Peak	End	Max Drawdown
*Cuban Missile Crisis*						
Tobacco	1959/12	1961/11	13	1961/12	1963/11	-44%
*Vietnam War*						
Personal Services	1966/06	1968/05	12	1968/12	1970/05	-69%
Real Estate	1966/06	1968/05	24	1969/06	1970/05	-52%
*End of Bretton Woods–Oil Crisis*						
Restaurants&Hotels	1970/07	1972/06	40	1973/01	1974/06	-55%
Entertainment	1970/06	1972/05	24	1973/01	1974/05	-60%
*Volcker Bear–‘Stagflation’*						
Precious Metals	1978/01	1979/12	14	1980/10	1981/12	-48%
Petro&Natural Gas	1978/11	1980/10	272	1980/12	1982/10	-60%
Construction	1978/11	1980/10	51	1980/12	1982/10	-64%
*Dot-com Bubble*						
Computer Software	1997/04	1999/03	551	2000/03	2001/03	-60%
Meas.&Contr. Equipm.	1998/03	2000/02	127	2000/03	2002/02	-61%
Electronic Equipment	1998/01	1999/12	347	2000/04	2001/12	-75%
Computer Hardware	1997/04	1999/03	190	2000/09	2001/03	-68%
Steel Works	1998/09	2000/08	77	2000/09	2002/08	-66%
*Housing Bubble*						
Steel Works	2005/06	2007/05	48	2008/06	2009/05	-75%
Coal	2006/07	2008/06	13	2008/07	2010/06	-74%

The Table lists the identified price run-ups of Fama-French 49 industries that experienced a crash during the subsequent 2 years during a period 1959–2014. A run-up is defined as any incident with (1) 100% raw and value-weighted return over the past two years (2) 100% net-of-market returns over the past two years, and (3) 100% raw return over the past five years. A crash is defined as a 40% drawdown from any point in the two years after the initial price run-up. We document the start and end date for all price run-ups, the number of firms in an industry at the time of the price run-up, the month of the price peak and the end date of the subsequent 2-years period and the maximal price drawdown within that period. Crash episodes are categorized based on particular S&P500 bear markets.

**Table 2:** Industry price run-ups–non-crashes.

	Run-up period			Subsequent 2-years		
Industry Name	Run-up Start	Run-up End	Number of Firms	Start	End	Max Drawdown
Aircraft	1963/12	1965/11	31	1965/12	1967/11	-26%
Industrial Mining	1964/02	1966/01	31	1966/02	1968/01	-28%
Meas.&Contr. Equipm.	1965/05	1967/04	28	1967/05	1969/04	-21%
Construction	1965/07	1967/06	19	1967/07	1969/06	-26%
Entertainment	1965/07	1967/06	27	1967/07	1969/06	-17%
Restaurants&Hotels	1965/12	1967/11	23	1967/12	1969/11	-29%
Aircraft	1974/10	1976/09	33	1976/10	1978/09	-19%
Healthcare	1976/05	1978/04	36	1978/05	1980/04	-12%
Computer Software	1976/09	1978/08	14	1978/09	1980/08	-25%
Healthcare	1978/05	1980/04	35	1980/05	1982/04	-28%
Computer Software	1990/11	1992/10	205	1992/11	1994/10	-8%
Textile	1990/11	1992/10	49	1992/11	1994/10	-21%
Recreation	1990/11	1992/10	44	1992/11	1994/10	-14%
Construction	2001/11	2003/10	51	2003/11	2005/10	-13%
Industrial Mining	2003/03	2005/02	14	2005/03	2007/02	-12%

The Table lists the identified price run-ups of Fama-French 49 industries that experienced no crash during the subsequent 2 years during a period 1959–2014. A run-up is defined as any incident with (1) 100% raw and value-weighted return over the past two years (2) 100% net-of-market returns over the past two years, and (3) 100% raw return over the past five years. The ‘subsequent 2 years’ are the two years after the initial price run-up. We document the start and end date for all price run-ups, the number of firms in an industry at the time of the price run-up, the start and end date of the subsequent 2 years period and the maximal price drawdown within that period.

In Table 1, I classify crash episodes into categories based on particular S&P500 bear markets that I identified earlier. Interestingly, all industry crashes, identified by Greenwood et al. [1], are coinciding with notable bear markets. Therefore, industry price run-ups can be of idiosyncratic nature, but industries are crashing, because of market-wide stress. One can think about two different scenarios here: (1) initially, the risk is more industry-specific, but because e.g. the adoption of a new technology, it gradually changes from idiosyncratic to systematic; (2) or in case of a macroeconomic shock, e.g. oil price shock, the risk changes from macro (macroeconomic/political) risk to systematic risk.

### Bubble episodes

**Fig 2:**
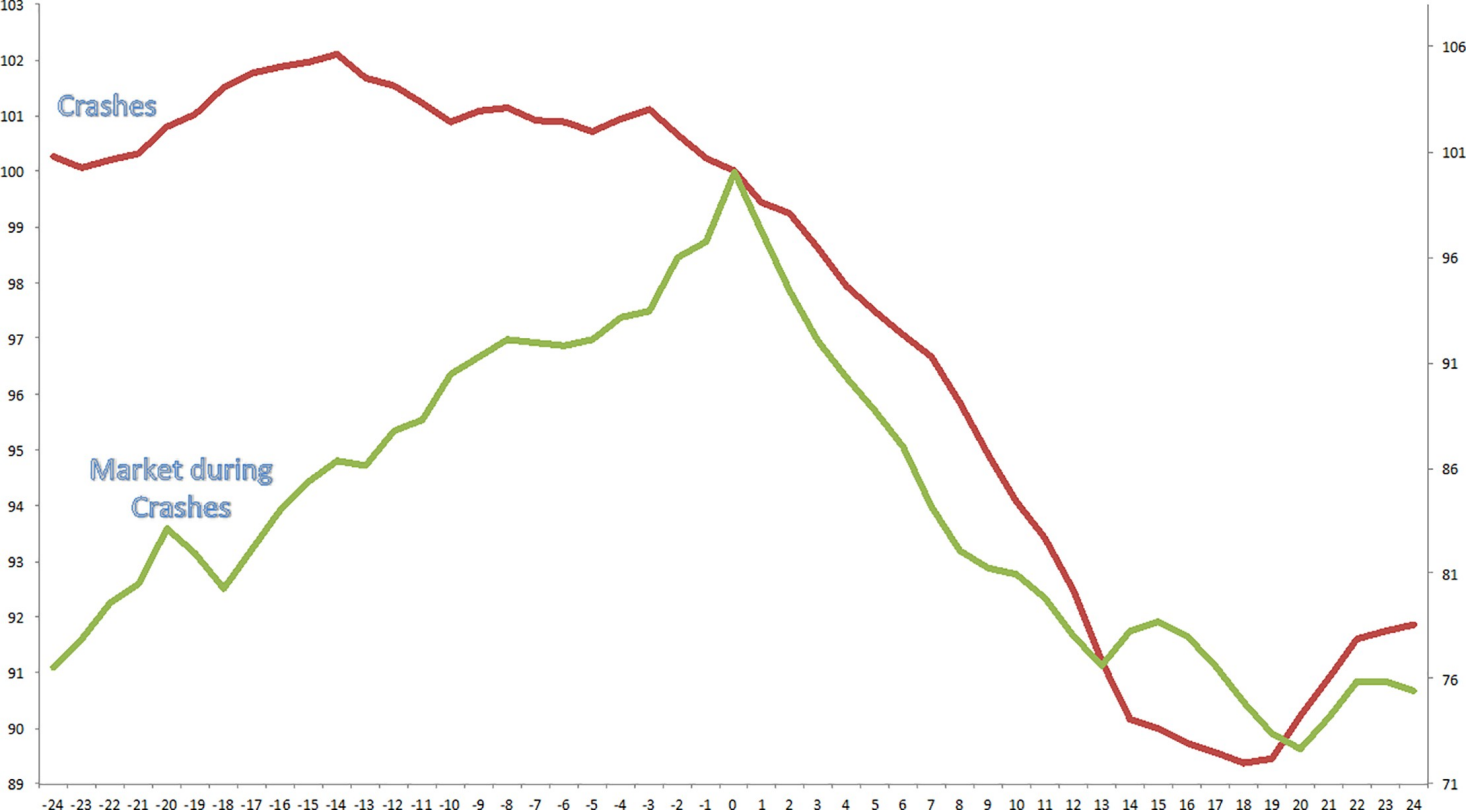
Fear and Euphoria during crashes (1959–2014). The Figure plots cumulative returns to the Euphoria Index EIx (left axis) and the S&P500 (right axis) during periods when US industries experience a large price run-up and a subsequent crash. The sample includes all industries between 1959 and 2012 and is based on Fama and French [39] 49-industry classifications. 30 episodes are identified in which an industry experiences both a raw and net-of-market return of 100% in a two-year period, and a raw return of 50% or more in a five-year period. 15 of these 30 episodes experienced a subsequent crash, and 15 episodes did not. A crash is defined as a 40% drawdown from any point in the two years after the initial price run-up. In the figure’s horizontal axis, 0 denotes the period in which an industry experiences a price peak and subsequently crashes. Both total return indices are normalized to have a value of 100 at this time.

In the following, I analyze the particular euphoria-fear-pattern in relation to the industry crashes that I identified. Average results are presented and particular examples are discussed. In Fig 2, I plot cumulative returns to the Euphoria Index EIx and the S&P500 during periods when US industries experience a large price run-up and a subsequent crash. In the figure’s horizontal axis, 0 denotes the period in which an industry experiences a price peak and subsequently crashes. Both total return indices are normalized to have a value of 100 at this time. It shows that Greenspan’s central claim holds up in the US industry data. Initially, euphoria builds up until, on average, 14 months before the crash. The increase in euphoria in that period is statistically significant at the 5% level. Subsequently, euphoria turns in to fear, first moderately, but a quarter before the crash fear increases sharply, which is, again, statistically significant at the 5% level. What is surprising here is that the turning point is already long before the crash. After ~18 months after the crash, investors become euphoric again. The pattern of market returns is more straightforward; the market increases before the crash and decreases after the crash, which relates to the strong market component of industry crashes discussed earlier.

**Fig 3:**
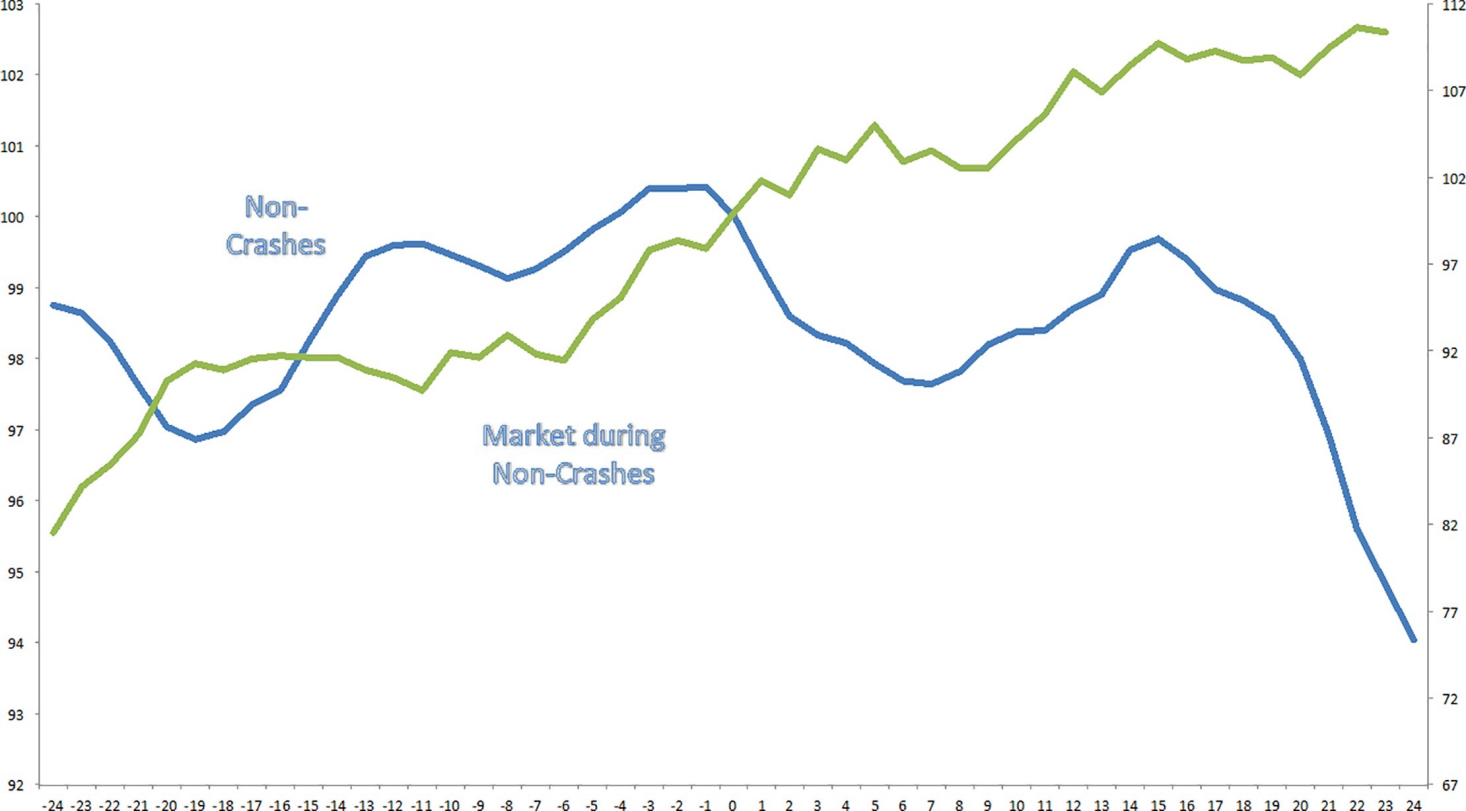
Fear and Euphoria during non-crashes (1959–2014). The Figure plots cumulative returns to the Euphoria Index EIx (left axis) and the S&P500 (right axis) during periods when US industries experience a large price run-up and do not crash afterwards. The sample includes all industries between 1959 and 2012 and is based on Fama and French [39] 49-industry classifications. 30 episodes are identified in which an industry experiences both a raw and net-of-market return of 100% in a two-year period, and a raw return of 50% or more in a five-year period. 15 of these 30 episodes experienced a subsequent crash, and 15 episodes did not. A crash is defined as a 40% drawdown from any point in the two years after the initial price run-up. In the figure’s horizontal axis, 0 denotes the period in which a price run-up was first observed. Both total return indices are normalized to have a value of 100 at this time.

In Fig 3, I plot cumulative returns to the Euphoria Index EIx and the S&P500 during periods when US industries experience a large price run-up and do not crash afterwards. Again, the figure’s horizontal axis, 0 denotes the period in which an industry experiences a price peak and subsequently crashes. Both total return indices are normalized to have a value of 100 at this time. It contrast to crashes, it can be observed that the particular euphoria-fear-pattern does not exist. Indeed, euphoria builds up in the second half of the run-up phase, but in the subsequent 2 years no particular pattern emerges. Only 15 months after the run-up phase euphoria clearly turns into fear. Again, the pattern of market returns is more straightforward; the market increases constantly during the 4-years period. Interestingly, the completely different pattern in the EiX that I find in last year before the crash or non-crash suggests, that crashes might be predictable ex ante.

**Fig 4:**
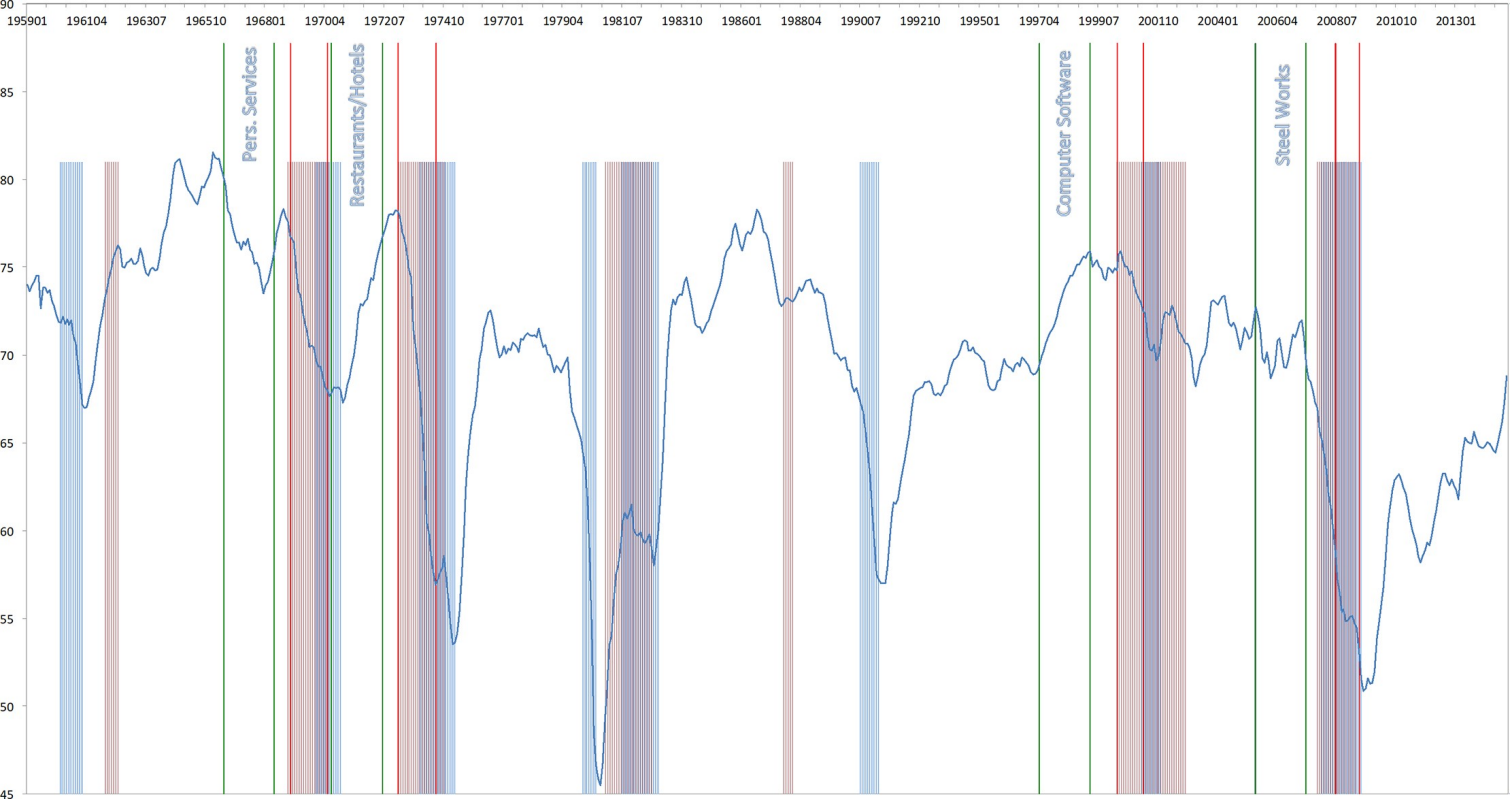
Euphoria Index and run-up/crash periods (1959–2014). The Figure depicts the level of the Euphoria Index during selected run-up and subsequent crash episodes for a time period 1959 until 2014. The highlighted areas present the NBER recession periods (in blue) and the S&P 500 bear markets (in red). For each bubble episode, the two green bars indicate the start and the end of the run-up period. The subsequent two red bars indicate the start and the end of the crash period. For each run-up/crash episode, the industry is mentioned.

**Fig 5:**
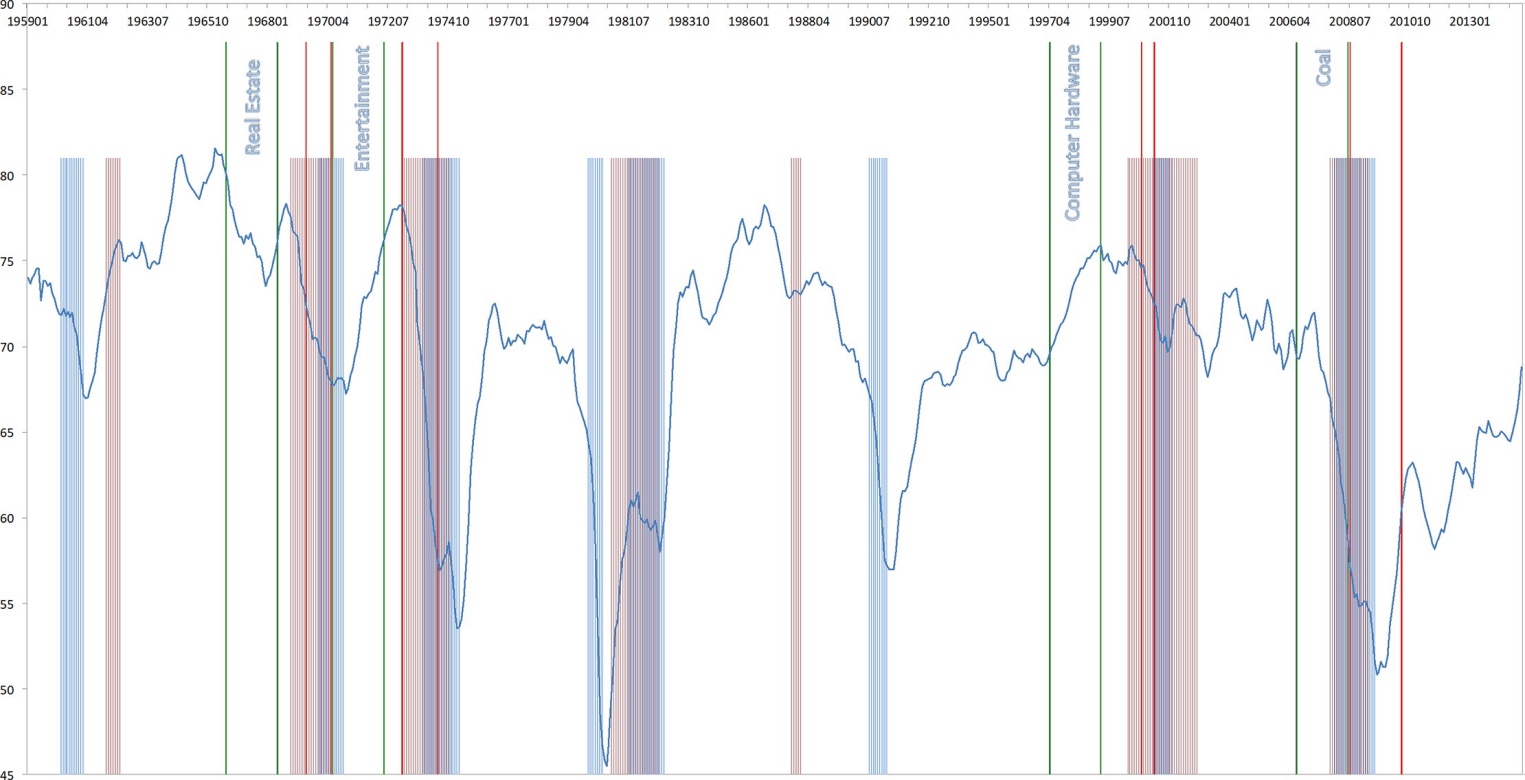
Euphoria Index and run-up/crash episodes (1959–2014). The Figure depicts the level of the Euphoria Index during selected run-up and subsequent crash episodes for a time period 1959 until 2014. The highlighted areas present the NBER recession periods (in blue) and the S&P 500 bear markets (in red). For each bubble episode, the two green bars indicate the start and the end of the run-up period. The subsequent two red bars indicate the start and the end of the crash period. For each run-up/crash episode, the industry is mentioned.

In the following, I discuss various bubble episodes and analyze the particular euphoria-fear-patterns that I observe. In Figs 4 and 5, I plot the level of the Euphoria Index during selected run-up and subsequent crash episodes for a time period 1959 until 2014. The highlighted areas present the NBER recession periods (in blue) and the S&P 500 bear markets (in red). For each bubble episode, the two green bars indicate the start and the end of the run-up period. The subsequent two red bars indicate the start and the end of the crash period. For each run-up/crash episode, the industry is mentioned.

#### Example: Vietnam war

Around the end of the 1960’s, the pronounced growth period turned into a mild recession with an annual inflation of about 6 percent. Due to the U.S. involvement in Vietnam, the national atmosphere was quite stressed. Around November 1968, together with the election of Richard Nixon, the stock market suffered a sharp drop. Regarding the industry crashes in that period, one can observe that the initial fear during the run-up phase in the ‘Personal Services’ and ‘Real Estate’ industry, turned into euphoria, which peaked in September 1968. From October 1968 onwards, investors became fearful, quite some months before we observe the crashes in the ‘Real Estate’, but also ‘Personal Services’ industries.

#### Example: Oil crisis

In the early 1970’s, Israel's Yom Kippur War and the subsequent Arab oil embargo resulted in a substantial increase of energy prices, hence, high inflation and a long-lasting recession. President Nixon resigned in August 1974 due to the Watergate scandal. Over a period from January 1973 to October 1974, the S&P 500 experienced a sharp drop of 48 percent. In this environment, the ‘Entertainment’ and ‘Restaurants & Hotels’ industries initially experienced a price run-up in an euphoric market, which was cooling down during the second half of 1972, and both industries crashed in January 1973, in a severe fearful environment, which lasted until February 1975.

#### Example: Dot-com bubble

In March 2000, the bursting dot-com bubble was the end of a period characterized by extraordinary speculation on new technology firms. The Nasdaq composite index experienced a sharp drop of 50 percent in nine months. Pastor and Verones [13]) studied the particular bubble episode. They argue that the risk associated with new technologies is initially mostly idiosyncratic. Once a technology is adopted, the risk gradually changes from idiosyncratic to systematic. The resulting increase in systematic risk depresses stock prices, because it pushes up the discount rates. In the spirit of Alan Greenspan, one could hypothesize that with the increasing probability of adaption of the new technology, ‘a bubble goes up slowly as euphoria builds’. When systematic risk increases, ‘fear hits and it comes down very sharply’. In Fig 4, I show the bubble episode in the ‘Computer Software’ industry and in Fig 5, the ‘Computer Hardware’ industry. The graph suggests that what Greenspan describes is exactly the pattern that I observe. During the run-up phase euphoria builds, but turns into fear already before the crash. After the crash, fear really hits. The pattern can be observed for all 5 industry crashes during the dot.com bubble.

#### Example: Housing bubble

In 2007, the subprime mortgage crisis became a nationwide financial crisis, that contributed to a severe recession. It was triggered by a large decline in home prices after the collapse of a housing bubble, leading to mortgage delinquencies, foreclosures, and the devaluation of housing-related securities. During the same period, oil and other commodity markets were characterized by enormous price increases. In 2008, the rhodium market experienced a bubble with enormous price increases until July 2008 and sharp price decreases until January 2009. During 2005 and 2007, soaring uranium prices led to high market values for uranium mining and exploration companies. Another price bubble that burst during 2008. In this environment, the ‘Steel Works’ and ‘Coal’ experienced a price run-up until June 2008 in a partly euphoric and partly fearful environment. Until the industries were crashing, fear was ‘hitting hard’ and continued to increase until summer 2009.

## Conclusions

The distinguished economist and former chairman of the Federal Reserve Alan Greenspan does believe that security prices exhibit price bubbles. In this paper, I evaluate Greenspan’s claim that stock price bubbles build up in periods of euphoria and tend to burst due to increasing fear. Indeed, there is evidence that e.g. during a crisis, triggered by increasing fear, both qualitative and quantitative measures of risk aversion increase substantially. It is argued that fear is a potential mechanism underlying financial decisions and drives the countercyclical risk aversion. Inspired by this evidence, I construct an euphoria/fear index, which is based on an economic model of time varying risk aversion. In line with the theoretical reasoning, results suggest that NBER recession periods are preceded by periods of increasing fear. The Greenwood et al. [1] methodology is used to identify periods of price run-ups and subsequent crashes for US industries 1959–2014. Interestingly, all industry crashes, are coinciding with notable bear markets. Therefore, industry price run-ups can be of idiosyncratic nature, but industries are crashing, because of market-wide stress. Furthermore, my findings suggest that (1) Greenspan is correct in that the price run-up initially occurs in periods of euphoria followed by a crash due to increasing fear; (2) on average already roughly a year before an industry is crashing, euphoria is turning into fear, while the market is still bullish; (3) there is no particular euphoria-fear-pattern for price-runs in industries that do not subsequently crash. I interpret the evidence as suggesting that Greenspan, who was labeled ‘Mr. Bubble’ by the *New York Times*, was not the serial ‘bubble blower’ that he was accused to be.
